# Denosumab’s Role in Reducing Type 2 Diabetes Risk and Improving Glycaemic Control in Osteoporotic Patients - A Meta-analysis

**DOI:** 10.5704/MOJ.2603.016

**Published:** 2026-03

**Authors:** R Ngo, D De-Jesus, C Azucena

**Affiliations:** Department of Orthopaedics, Jose R Reyes Memorial Medical Center, Manila, Philippines

**Keywords:** denosumab, osteoporosis, type 2 diabetes, risk reduction, bone health

## Abstract

**Introduction:**

Osteoporosis, common among postmenopausal women and the elderly, is primarily associated with increased fracture risk. Emerging evidence suggests a potential link between bone metabolism and glucose regulation with implications for type 2 diabetes (T2D) risk. Denosumab, a monoclonal antibody effective in increasing bone density, might also reduce the risk of T2D. This meta-analysis evaluates the impact of denosumab on T2D risk and glycaemic markers, including fasting blood sugar (FBS), HbA1c, and Homeostatic Model Assessment of Insulin Resistance (HOMA-IR).

**Materials and methods:**

A comprehensive search of PubMed, Cochrane Library, and Embase identified randomised controlled trials and cohort studies comparing denosumab with placebo or other osteoporosis treatments. Study quality was assessed using the Cochrane risk-of-bias tool. The primary outcome was T2D incidence, with risk ratios and related metrics analysed for consistency.

**Results:**

Results revealed a 22% reduction in T2D incidence with denosumab (HR = 0.78, 95% CI 0.70, 0.87, p<0.00001, I^2^= 62%) with small benefit in terms of absolute risk reduction (ARR = -0.01, 95% CI -0.03, 0.01, p = 0.19). Denosumab also resulted in small reductions in FBS in the included RCTs (SMD = -0.13, 95% CI -0.30, 0.03, p = 0.0798) and non-randomised trials (SMD = -0.28, 95% CI - 0.63, 0.06, p = 0.0947). There were slightly larger but still non-significant reductions in HbA1c (SMD = -0.44, p = 0.10) and HOMA-IR (SMD = -0.21, p = 0.114).

**Conclusion:**

Denosumab may confer metabolic benefits beyond bone health, particularly in reducing T2D risk and improving FBS. While this aligns with its role in modulating bone turnover and RANKL inhibition, inconsistent outcomes for HbA1c and HOMA IR underscore the need for further investigation into its glycaemic effects.

## Introduction

Osteoporosis, a condition characterised by low bone mass and structural deterioration of bone tissue, leads to an increased risk of fractures, particularly in postmenopausal women and the elderly^1^. Type 2 diabetes (T2D) has been increasingly recognised as a contributing factor to skeletal fragility, further complicating the clinical management of osteoporosis. Although T2D is generally associated with higher bone mineral density (BMD), it paradoxically increases fracture risk, suggesting that bone quality, rather than density alone, plays a critical role in fracture susceptibility among diabetic patients^2,3^. The mechanisms underlying this phenomenon are complex, involving factors such as advanced glycation end products (AGEs) that weaken bone collagen and impair bone turnover^4^.

Denosumab, a monoclonal antibody targeting the receptor activator of nuclear factor-kappa B ligand (RANKL), is widely used to treat osteoporosis by inhibiting osteoclast formation and function, thereby increasing BMD and reducing fracture risk^5^. Recent studies have suggested that denosumab may also provide metabolic benefits by lowering the risk of developing T2D, though the exact relationship between bone health and T2D remains unclear^6,7^. This emerging evidence points to a potential therapeutic intersection between bone and metabolic health, mediated in part by the RANK/RANKL/OPG signalling pathway, which has been implicated in both bone resorption and insulin resistance. Given the frequent coexistence of osteoporosis and T2D in aging populations, identifying treatment that addresses both conditions may help reduce polypharmacy, simplify care regimens, and enhance adherence, ultimately improving overall patient outcomes and reducing healthcare burden.

This meta-analysis aims to systematically evaluate the efficacy of denosumab in reducing the risk of T2D in patients with osteoporosis, as well as to explore its impact on glycaemic parameters. By synthesising the available evidence, we seek to determine whether denosumab offers a dual benefit in improving bone health and mitigating T2D risk.

## Materials and Methods

This meta-analysis was performed in accordance with the PRISMA 2020 statement. A comprehensive systematic search of PubMed, Embase, and the Cochrane Library was performed to identify relevant peer-reviewed articles published in English. The following keywords were used: risk reduction, osteoporosis, denosumab, diabetes, and bone health.

The inclusion criteria encompassed randomised controlled trials (RCTs) and cohort studies assessing the effect of denosumab on type 2 diabetes risk in adult patients (≥60 years) diagnosed with osteoporosis, including postmenopausal women and older adults. Eligible studies were required to focus on denosumab as the primary treatment, administered at 60mg subcutaneously, and compare it to placebo, no treatment, or other osteoporosis therapies (e.g., bisphosphonates, teriparatide, raloxifene). Included studies were required to report outcomes related to the incidence of new-onset type 2 diabetes or changes in glycaemic parameters such as fasting blood glucose, HbA1c, and HOMA IR during the follow-up period. Unlike fasting blood glucose and HbA1c, HOMA-IR provides information on insulin dynamics since it’s a validated surrogate marker of insulin resistance. HOMA-IR is calculated from fasting glucose and insulin concentrations and is widely used in clinical and epidemiological studies to assess early metabolic dysfunction and evaluate the risk of progression to T2D.

Only peer-reviewed articles published in English were considered. Conversely, studies were excluded if they were case reports, editorials, reviews, or commentaries; involved populations with metabolic disorders unrelated to osteoporosis; examined interventions other than denosumab or combined therapies; lacked relevant metabolic outcomes; or primarily focused on bone health without assessing glycaemic parameters. Additionally, non-English publications, abstracts, unpublished data, conference proceedings, and non-peer-reviewed sources were excluded.

Two authors independently screened and reviewed the abstracts and articles for inclusion. Articles were selected based on the inclusion criteria, and the decision to include the article was made through consensus. Initial database search with the search criteria yielded 2397 publications. After refining the search parameters, excluding studies that were not RCTs or Cohort studies, the search yielded 113 articles. Based on the title and abstract, a further 103 articles were found to have outcomes or study designs not compatible with the inclusion criteria, and these were excluded. Full texts of 13 studies were reviewed; of these, two involved concomitant therapies, and one focused on outcomes unrelated to glycaemic parameters. Ultimately, nine articles met the eligibility criteria and were included in the meta-analysis. Studies with incompatible comparison groups or outcomes were excluded.

Data were extracted from articles with reported outcomes on the effects of denosumab on the incidence of diabetes mellitus type 2 and on the glycaemic control of diabetic individuals with osteoporosis. Values not presented in tabular form were abstracted from graphs and other visual aids using graphical software. Binomial/categorical data were tabulated in Microsoft Excel as frequencies, and continuous data (which included both change-from-baseline scores and values obtained at follow-up) as means and standard deviations. Tabulations of the latter were done for measurements obtained when available at 3 months, 6 months, and 12 months follow-up. Data on the incidence of DM Type 2 were extracted either as the number of incident cases or as hazard ratios (HRs), depending on availability. Data from Weivoda *et al* (2020) was entered twice due to their use of three study groups; first as denosumab vs bisphosphonates, and second as denosumab vs calcium/Vitamin D, which was considered as the placebo group^8^.

Data transformations for meta-analyses were performed using procedures outlined in Chapter 6 of the Cochrane Handbook for Systematic Review of Interventions. Whenever data was reported as means with 95% confidence intervals, the upper and lower bounds of the latter were used to derive estimates of the standard deviation, accounting for the sample sizes of their corresponding studies. All values of FBS were converted into mmol/L for uniformity.

For the incidence of diabetes, analysis was conducted using random effects models and a generic inverse variance approach. Effect measures, including risk ratios (RRs) and hazard ratios (HRs), were compared for consistency. For the analyses of glycaemic control (FBS, HbA1c, HOMA-IR), a three-level fixed effects meta-analytic model was used to create summary forest plots, one for each of the subsets of randomised controlled trials, and for non-randomised clinical trials. Separate analyses were conducted using all available data to account for multiple measurements present within each study followed by moderator analysis with the following dummy coded variables: follow-up time (3 months vs 6 months vs 12 months); comparator group (placebo vs bisphosphonates vs baseline levels [for repeated measure designs]; and study design (RCT vs before-after trials vs retrospective cohort). The resulting effect sizes are presented as standardised mean differences (SMDs) with 95% confidence intervals (CI). Heterogeneity was estimated using Chi square and Higgins’ I2 statistic, with values classified as minimal (0-40%), moderate (30-60%), substantial (50-90%), or considerable (75-100%), as per Cochrane guidelines. Sources of heterogeneity for three-level meta-analytic models were divided into different measurements within the same study (Level 2) and between measurements from different studies (Level 3). Statistical significance was tagged at p = 0.05. Cochrane Review Manager (RevMan) 5 and R Statistical Software (v4.4.0) with the metaphor package were used for the computation of effect measures and the creation of forest plots.

Risk of bias in RCTs was assessed using the Revised Cochrane Risk-of-Bias Tool for Randomized Trials (RoB 2), which evaluates five domains: (1) bias arising from the randomisation process, (2) deviations from the independent interventions (effect of assignment to intervention and effect of adhering to intervention), (3) missing outcome data, (4) measurement of the outcome, and (5) selection of the reported result. The counterpart for non-randomised trials, including repeated measures studies, is the Risk of Bias in Non-Randomised Studies of Interventions (ROBINS-I) tool. This tool evaluates similar domains, with adaptations for confounding, participant selection, and intervention classification. Domains were graded as high, low, or with some concerns. Overall assessments of risk of bias for each article were then decided based on the grading of each section. Post Hoc re-analysis of previously published trial results, which included Napoli *et al* and Schwartz *et al* were no longer assessed for risk of bias^9,10^.

Analyses were conducted using random effects models and a generic inverse variance approach for incidence of diabetes. The resulting effect measures of risk ratios and hazard ratio were compared for consistency. A three-level fixed effects meta-analytic model was used to create summary forest plots analysing all available data for the analysis of glycaemic control to account for multiple measurements present within each study (for different follow-up times, and for different comparator groups) as well as for moderator analysis with follow-up time and comparator group used. The latter were dummy coded for use in the meta-regression model, with the following levels: 3 months, 6 months and 12 months for follow-up time; and placebo, bisphosphonates, and baseline levels (in repeated measures studies) for the comparator group. The resulting effect sizes are presented as standardised mean differences with 95% confidence intervals. Heterogeneity was estimated using Chi square and Higgin’s I2statistic, with values classified as minimal, moderate, substantial and considerable for scores of 0-40%, 30-60%, 50-90%, and 75-100% respectively as patterned from the values used in the Cochrane Handbook. Statistical significance was tagged at p = 0.05. All computations of effect measures and forest plot generation were performed using Cochrane Review Manager (RevMan) 5 and R Statistical Software (v4.4.0) with the *metafor* package.

## Results

An initial search using the search criteria previously outlined in the methodology yielded 2397 articles, which were reduced to 113 once the criteria for inclusion in the present study were applied. Manual screening of titles and abstracts resulted in the exclusion of several articles due to having outcomes unrelated to the present study, or study designs incompatible with the analysis. The last phase of screening via reading the full texts resulted in the exclusion of 3 were found to have the concomitant use of other therapies which did not fit the inclusion criteria. Data was still extracted from these articles for the review and discussion but were excluded in the meta-analysis.

**Fig. 1:**
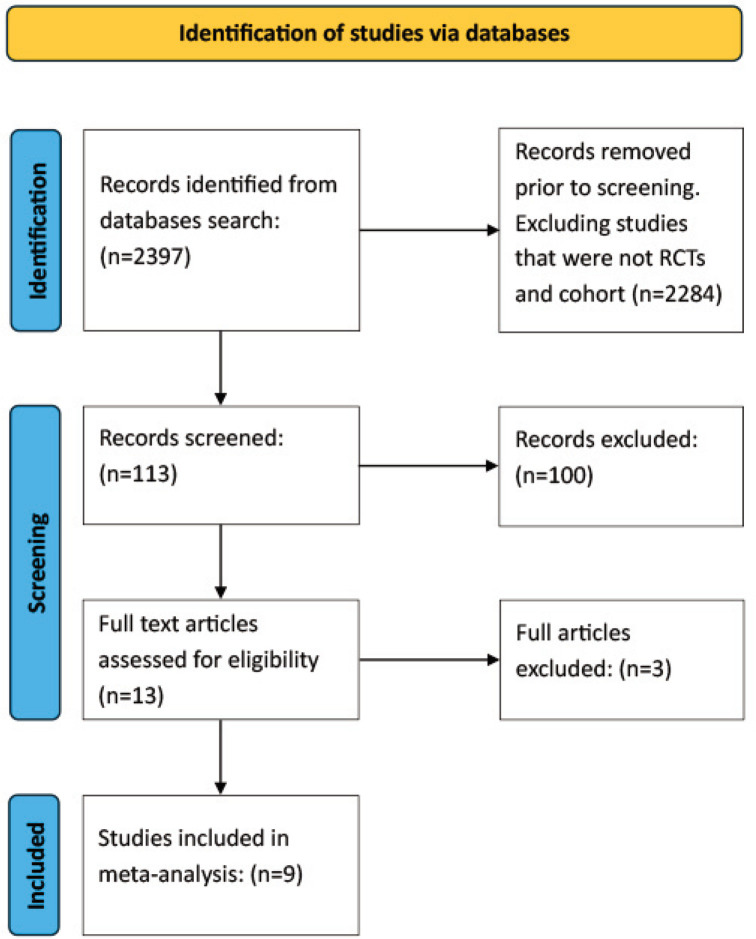
PRISMA flowchart summarising the study selection process. A total of 2,397 records were identified through database searches. After removing 2,284 studies that were not RCTs and cohort, 113 records were screened. Thirteen full-text articles were assessed for eligibility, and nine studies were ultimately included in the meta-analysis.

Fig. 1 presents the PRISMA flow diagram outlining the three phases of study selection, resulting in a final set of nine articles comprising a total study population of 172,385.

**Fig. 2:**
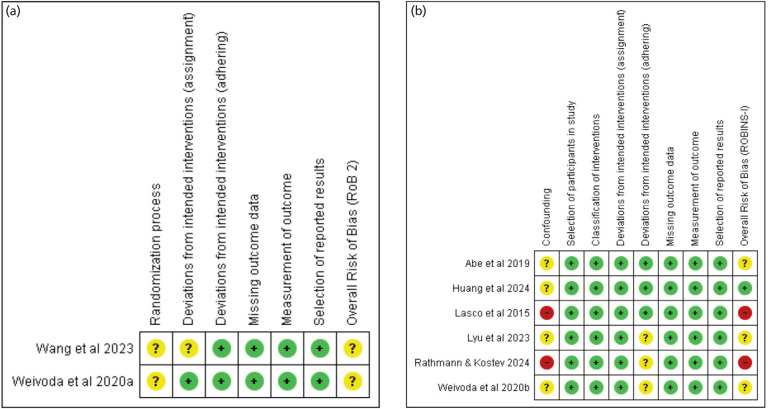
(a) Risk of bias assessments for randomised controlled trials using the revised Cochrane Risk-of-Bias tool for randomised trials (RoB 2) tool. (b) Risk of bias assessments for non-randomised clinical studies using the Cochrane Risk of Bias in non-randomised studies – of interventions (ROBINS-I) tool.

Characteristics of the studies included in the meta-analysis are tabulated in Table I^6-14^, outlining the intervention, control, and outcomes explored by each. Fig. 2 shows the risk-of-bias assessment. Slightly differing criteria were used for the assessment of randomised and non-randomised clinical trials, but there is some overlap of domains. Napoli *et al* and Schwartz *et al* were no longer assessed due to being a reanalysis of the data of previously published trials, rendering the assessment criteria inappropriate^9,10^. The randomised controlled trials of Weivoda *et al* (2020a) and Wang *et al* (2023) were generally at low risk of bias, except for certain domains due to the lack of documentation of the process of randomisation and allocation of participants in both, and the open-label nature of the study of Wang *et al*^8,11^. The uncontrolled, repeated measures studies of Lasco *et al* and Abe *et al* were at risk of bias due to possible confounding, although the latter had some slight mitigation with the control of medications prior to the start of administration of denosumab^12,13^.

**Table I:** Study characteristics of included studies.

Authors	Location and Recruitment Period	No. of Participants	Year	Methodology	Intervention	Placebo / Control	Follow-up	Outcome Measures
Abe *et al*^13^, 2019	Nagasaki, Japan	n = 20	2013-2018	Cohort	Denosumab 60mg SQ	None	52 weeks	Incidence of T2DM FBS, HBA1c, HOMA-IR,
Huang *et al*^6^, 2024	Taiwan	n = 101,296	2012-2019	Cohort	Denosumab 60mg SQ	Denosumab 60mg SQ but discontinued	1.9 years	Incidence of T2DM
Lasco *et al*^12^, 2015	Italy	n = 48	2012-2013	Cohort	Denosumab 60mg SQ	None	24 weeks	HOMA-IR
Lyu *et al*^14^, 2023	UK	n = 25339	1995-2021	Cohort	Denosumab 60mg SQ	Oral bisphosphonates	5 years	Incidence of T2DM
Napoli *et al*^9^, 2018	Italy	n = 7808	2018	RCT	Denosumab 60mg SQ	Placebo	3 years	ncidence of T2DM, FBS
Rathman and Koztev *et al*^7^, 2024	Germany	n = 30422	2010-2021	Cohort	Denosumab 60mg SQ	Alendronate	2 years	Incidence of T2DM
Schwartz *et al*^10^, 2013	USA	n = 7076	2013	Cohort	Denosumab 60mg SQ	Placebo	3 years	Incidence of T2DM, FBS
Wang *et al*^11^, 2023	China	n = 90	2021	RCT	Denosumab 60mg SQ	Ibandronate	6 months	FBS, HBA1c
Weivoda *et al*^8^, 2020^a^	USA	n = 56	2020	RCT	Denosumab 60mg SQ	Placebo	12 months	FBS, HBA1c
Weivoda *et al*^8^, 2020^b^	USA	n = 230	2020	Cohort	Denosumab 60mg SQ	Bisphosphate	12 months	FBS, HBA1c, HOMA-IR

The remainder, which were mostly analyses of medical records and databases, generally had some degree of control of baseline characteristics, whether via matching of participants or use of propensity score matching, except for Rathmann and Kostev^6-8,14^. Furthermore, only Huang *et al* were able to control for a broad list of possible covariates in their analysis, thus making them the only ones at low risk of bias for the domain of adherence to interventions^6^.

Nine studies were included in the meta-analysis examining denosumab’s effect on T2D incidence and glycaemic control^6-14^. Three of these reported on the incidence of diabetes mellitus type 2 in osteoporotic patients, while the rest reported on glycaemic control in osteoporotic patients already diagnosed with type 2 diabetes. Outcomes explored included fasting blood sugar, HbA1c, and HOMA-IR reported at 3 months, 6 months, and 12 months follow-up.

**Fig. 3:**
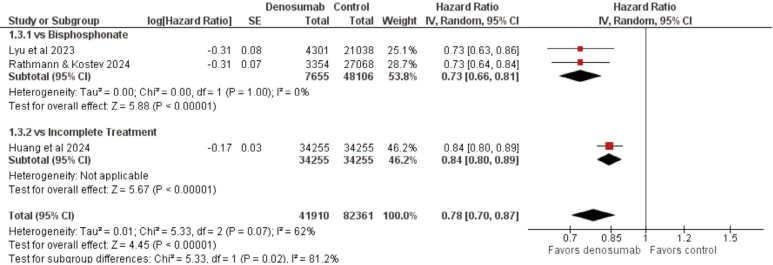
Forest plot of incidence of diabetes mellitus type 2 analysed as hazard ratio in denosumab vs control groups.

Fig. 3 shows the analysis of diabetes mellitus incidence using hazard ratios derived from each study’s individual adjusted Cox regression model (3 studies, 124,271 participants; HR = 0.78, 95% CI 0.70, 0.87, p<0.00001), which showed a 22% reduced risk of developing diabetes in the denosumab groups compared to control groups^6,7,14^. These results are consistent with analysis using ratios derived from the frequency of incident cases without considering the time component, which includes data from Schwartz *et al* (4 studies, 131,184 participants; RR = 0.77, 95% CI 0.62, 0.95, p = 0.02)^6,7,10,14^. The observed benefit in terms of absolute risk reduction however, is very small (ARR = -0.01, 95% CI - 0.03, 0.01, p = 0.19) which is due to the low incidence of diabetes observed in the included studies, both in treatment [2,265/45,357 (4.99%)] and control groups [4,729/85,827 (5.51%)]. Factors contributing to this include short follow-up times, attrition and loss to follow-up, missing data, and difficulties in accurately determining the presence or absence of diabetes retrospectively. Moderate to substantial heterogeneity was found in the analysis with hazard ratios (I2 = 62%, p = 0.07), which is explored more in the subsequent paragraphs.

Dividing the included articles into subgroups showed a much stronger reduction in the hazard (specifically by 27%) when comparing denosumab vs bisphosphonate groups (HR = 0.73, 95% CI 0.66, 0.81, p<0.00001), as compared to denosumab vs placebo (HR = 0.84, 95% CI 0.80, 0.89, p<0.00001). Inter-subgroup heterogeneity was also considerable (I2 = 81.2%, p = 0.02), indicating that the nature of the comparator group contributed heavily to the differences between results.

There are important considerations and caveats for this analysis: the sole study that compares denosumab vs placebo defined their simulated placebo group as participants who received an initial dose of denosumab but discontinued treatment, specifying that one dose was not expected to have sustained clinical effects^6^. Of the three included studies, only Rathmann and Kostev did not make use of propensity score matched samples, although this is mitigated by the use of multiple covariates during analysis, resulting in a hazard ratio adjusted for the effects of many possible confounders^7^. Use of adjusted hazard ratios resulted in a slightly weaker but significantly more precise effect size compared to the unadjusted analysis (adjusted: HR = 0.78, 95% CI 0.70, 0.87 vs unadjusted: HR = 0.76, 95% CI 0.59, 0.97). Possible variations in the effect according to subpopulation cannot be summarised across studies due to insufficient information; such interaction effects may be significant, as shown by the sensitivity analysis done by Lyu *et al* showing a 46% hazard reduction in prediabetics, which is much higher compared to the 8% reduction found in non-prediabetics^14^.

**Fig. 4:**
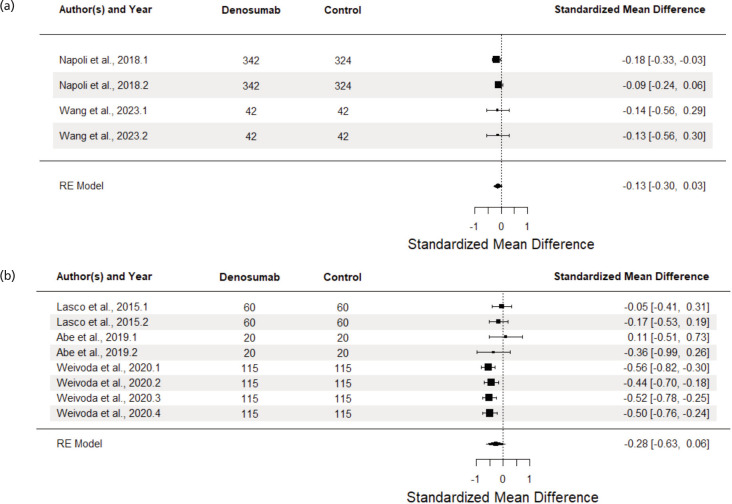
Forest plot of effect of denosumab vs control groups on fasting blood sugar in osteoporotic patients diagnosed with diabetes mellitus type 2 in (a) RCTs and (b) non-RCTs.

Fig. 4 shows FBS analysis, with non-RCTs showing larger, but nonsignificant reductions in denosumab vs comparator groups. Non-RCT studies showed a larger effect size (SMD = -0.28, 95% CI -0.63, 0.06, p = 0.0947) compared to RCTs (SMD = -0.13, 95% CI -0.30, 0.03, p = 0.0798). While heterogeneity in RCT studies was minimal (Q (3) = 0.60, p = 0.897, I2 = 0%), the opposite was found for the non-RCTs (Q (7) = 10.61, p = 0.1566, I2 = 62.19%), indicating variability between studies. Analysis of sources of heterogeneity of the latter showed minimal intra-study heterogeneity (I2Level2 = 0%) but moderate to substantial inter-study heterogeneity (I2Level3 = 62.19%). This indicates that the variability mostly stems from differences between studies, which may be due to differences in methodology and patient populations used.

Moderator analysis was conducted using all available data, regardless of study design. With follow-up as a moderator variable, slightly stronger effects were seen at increasingly larger intervals since study initiation, but there was insufficient evidence to conclude statistical significance (F (2,9) = 0.42, p = 0.667). Similar results were found for the analysis of the type of comparator group as moderator (F (2,9) = 0.27, p = 0.77). On the other hand, significant differences were found when comparing between study designs (F (2,9) = 10.32, p = 0.0047): RCTs and repeated measure studies did not significantly differ from one another, but non-randomised studies showed significantly stronger reductions in FBS by an average of 0.37 compared to RCTs (95% CI -0.56, -0.18, p = 0.0019).

**Fig. 5:**
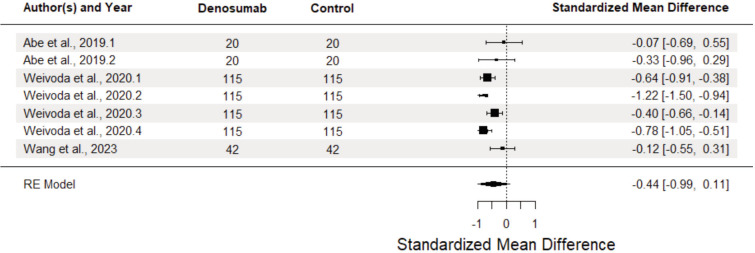
Forest plot of effect of denosumab vs control groups on HbAIc in osteoporotic patients diagnosed with diabetes mellitus type 2.

Fig. 5 shows HbA1c analysis, which revealed no significant differences (SMD = -0.44, 95% CI: -0.99 to 0.11, p = 0.10), with considerable heterogeneity (I^2^= 84.97%). However, there was considerable heterogeneity (I2=84.97%, p<.0001), which came equally from differing results within studies and across (I2Level2 = 40.66%, I2Level3 = 44.31%). Data from the subset of non-randomised clinical trials showed a stronger but still non-significant reduction (SMD = -0.54, 95% CI -1.24, -.16, p=0.10) compared to the sole RCT.

Moderator analysis revealed no significant differences in effect sizes based on follow-up duration, (F (2,4) = 2.60, p=0.189), comparator group (F (2,4) = 0.50, p = 0.64), and study design (F (2,4) = 1.88, p = 0.2659), suggesting that effects did not systematically differ according to time, comparison group or study design, at least for the included studies. Similar to the analysis for FBS, slightly stronger effects on average were seen at later time-points.

**Fig. 6:**
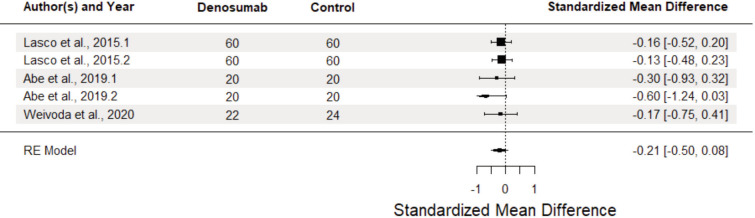
Forest plot of effect of denosumab vs control groups on HOMA-IR in osteoporotic patients diagnosed with diabetes mellitus type 2.

Fig. 6 shows HOMA-IR analysis, with no significant reduction (SMD = -0.21, 95% CI: -0.50 to 0.08, p = 0.114). Heterogeneity was minimal (I2 = 0%, p=0.762), indicating that all studies unanimously found non-significant results. Limiting the analysis to the subset of non-randomised studies resulted in a stronger but more imprecise estimate of effect (SMD = -0.24, 95% CI -0.69, 0.22, p = 0.19), as well as greater heterogeneity, specifically inter-study heterogeneity (I2Level3 = 19.22%). Moderator analysis was no longer performed due to the small number of observations present.

## Discussion

This meta-analysis investigated the potential role of denosumab in reducing the incidence of T2D and improving glycaemic control in osteoporotic patients. Our findings demonstrated that denosumab was associated with a statistically significant 22% reduction in the risk of developing T2D. The strength of this result lies in the use of effect measures adjusted for multiple possible confounders via propensity score matching and/or addition of covariates to the Cox regression models. Additionally, a small to moderate reduction in fasting blood sugar (FBS) was observed among patients with T2D, while no significant effects were found for HbA1c or HOMA-IR.

These findings align with previous hypotheses and studies investigating the role of RANKL inhibition in metabolic regulation. Napoli *et al* hypothesised that RANKL may influence insulin sensitivity through inflammatory pathways, and preclinical studies have supported this notion by demonstrating improved glucose homeostasis with RANKL inhibitors^9^. Similarly, Schwartz *et al* reported trends toward improved metabolic outcomes with denosumab, although their reanalysis did not include sufficient data for conclusive results^10^. The reduction in FBS observed in our analysis aligns with the findings of Lasco *et al* and Abe *et al*, who suggested a possible impact of denosumab on fasting glucose levels through reduced inflammatory cytokine activity^12,13^.

However, our results differ from Huang *et al*, who found a stronger association between denosumab and reductions in HbA1c^6^. This discrepancy could be attributed to differences in study populations and the breadth of covariates adjusted for in their analysis. Our meta-analysis, which included studies with diverse populations and methodologies, observed considerable heterogeneity in HbA1c outcomes, with no statistically significant effect overall.

The absence of significant effects on HbA1c and HOMA-IR in our study may be attributed to several factors. First, many included studies assessed outcomes at 3-, 6-, and 12-month intervals, which may be insufficient to detect significant changes in long-term glycaemic markers such as HbA1c. HbA1c reflects average glucose levels over approximately 8 to 12 weeks and is less sensitive to short-term metabolic changes than markers like FBS. Furthermore, some studies included patients with well-controlled sugar at baseline, reducing the potential for further improvement and introducing a ceiling effect. Additionally, the limited sensitivity and accuracy of HOMA-IR, particularly with established T2D or in observational settings, may have contributed to the non-significant findings. Moreover, the use of concomitant glucose-lowering medications may have confounded glycaemic outcomes and masked any additive effects of denosumab.

Despite these limitations, the biological plausibility of denosumab’s metabolic benefits remains strong. The receptor activator of nuclear factor kappa-B ligand (RANKL) is a key regulator of osteoclastogenesis, with emerging evidence implicating its involvement in glucose metabolism and insulin resistance^15^. Understanding the metabolic effects of RANKL inhibition, particularly through agents like denosumab, opens new avenues in the management of T2D alongside osteoporosis.

RANKL binds to its receptor, RANK, activating signalling pathways such as nuclear factor kappa B (NF-κB), which is integral to inflammatory and metabolic processes. Chronic activation of these pathways has been linked to increased systemic inflammation and insulin resistance. Pro inflammatory cytokines, including TNF-α and IL-6, which are downstream products of RANKL activation, impair insulin signalling by disrupting insulin receptor substrate-1 (IRS-1) activity and reducing glucose uptake in skeletal muscle and adipose tissue^16^. Additionally, RANKL may influence glucose homeostasis by interacting with metabolic tissues. For instance, RANKL signalling in adipocytes and skeletal muscle cells may alter mitochondrial function, promoting metabolic inefficiency and lipotoxicity, both of which contribute to insulin resistance^17^.

From a clinical standpoint, the findings of this meta-analysis suggest that denosumab may offer metabolic benefits in addition to its established efficacy in reducing fracture risk. However, variability and modest effect sizes observed in glycaemic outcomes indicate that these benefits should not be overstated. Instead, they may be considered an added value in appropriately selected patients. Specifically, individuals with osteoporosis who are also prediabetic or at high risk for developing T2D may derive dual benefits from denosumab therapy.

Denosumab, a monoclonal antibody that inhibits RANKL, reduces bone resorption while potentially exerting systemic anti-inflammatory effects that favour insulin sensitivity. Some studies have reported modest improvements in glycaemic indices such as FBS and HbA1c in patients receiving denosumab^8,11^. These effects may be mediated in part through the suppression of systemic inflammation and the modulation of osteokines, bone-derived hormones like osteocalcin, that are implicated in glucose homeostasis^18^.

Comparatively, other osteoporosis treatments such as bisphosphonates like alendronate and zoledronic acid have also demonstrated a lower risk of T2D. Although a large-scale meta-analysis noted non-significant reductions in diabetes incidence^19^. Compared to bisphosphonates, denosumab’s mechanism involves direct modulation of the RANKL pathway, which is more directly involved in systemic inflammation. Therefore, treatment decisions should be individualised, considering each patient’s fracture risk, metabolic profile, and comorbidities.

Our hypothesis that RANKL inhibition may represent a novel therapeutic approach in metabolic diseases is of great significance. By addressing the inflammatory and metabolic components of diabetes, denosumab could provide a dual benefit for osteoporotic patients at risk of or living with T2D. However, clinicians should be cautious in prescribing denosumab solely for metabolic benefit but rather consider these effects as a potential added value in selected patients.

The primary strength of this meta-analysis is the inclusion of diverse study designs and outcomes, allowing for a comprehensive evaluation of the effects of denosumab. Subgroup and moderator analyses further contextualise the findings across different comparator groups and study methodologies. However, the study has limitations, including significant heterogeneity in T2D incidence and glycaemic control outcomes, particularly HbA1c. The small number of T2D cases in individual studies led to wide confidence intervals, reducing precision. Furthermore, non-English publications were excluded, which may have introduced selection bias by omitting potentially relevant studies conducted in non-English-speaking regions. Most included studies also had limited follow-up durations, raising questions about the long-term effects of denosumab on metabolic outcomes.

Future research should focus on long-term, randomised controlled trials with larger sample sizes to confirm the metabolic effects of denosumab. Mechanistic studies investigating the interaction between RANKL inhibition and insulin sensitivity pathways would also enhance our understanding of its therapeutic potential. Additionally, subgroup analyses exploring patient-specific factors, such as baseline glycaemic status and concurrent medications, may help identify populations most likely to benefit from denosumab therapy.

## Conclusion

This meta-analysis suggests that denosumab may reduce the risk of type 2 diabetes mellitus and slightly improve fasting blood sugar levels in osteoporotic patients. However, its effects on glycaemic control markers such as HbA1c and HOMA-IR remain inconclusive. While the findings are promising, significant heterogeneity among studies underscores the need for further research to establish robust evidence and guide clinical practice. Denosumab appears to be a valuable therapeutic option for osteoporotic patients, particularly those with a higher risk of type 2 diabetes mellitus.
